# Prion protein E219K polymorphism: from the discovery of the KANNO blood group to interventions for human prion disease

**DOI:** 10.3389/fneur.2024.1392984

**Published:** 2024-07-10

**Authors:** Si-Si Wang, Zhao-Li Meng, Yi-Wen Zhang, Yi-Shuang Yan, Ling-Bo Li

**Affiliations:** ^1^Department of Translational Medicine, The First Hospital of Jilin University, Changchun, China; ^2^Aikang MedTech Co., Ltd., Shenzhen, China

**Keywords:** KANNO, E219K polymorphism, *PRNP*, prion protein, prion disease, alloantibody

## Abstract

KANNO is a new human blood group that was recently discovered. The KANNO antigen shares the *PRNP* gene with the prion protein and the prion protein E219K polymorphism determines the presence or absence of the KANNO antigen and the development of anti-KANNO alloantibodies. These alloantibodies specifically react with prion proteins, which serve as substrates for conversion into pathological isoforms in some prion diseases and may serve as effective targets for resisting prion infection. These findings establish a potential link between the KANNO blood group and human prion disease via the prion protein E219K polymorphism. We reviewed the interesting correlation between the human *PRNP* gene’s E219K polymorphism and the prion proteins it expresses, as well as human red blood cell antigens. Based on the immune serological principles of human blood cells, the prion protein E219K polymorphism may serve as a foundation for earlier molecular diagnosis and future drug development for prion diseases.

## 1 Introduction

The KANNO blood group system was confirmed as the 37th classification of the International Society of Blood Transfusion in August 2019 (1). The KANNO antigen is a human erythrocyte membrane protein that shares the encoding gene *PRNP* with prion protein (PrP) (2). The PrP E219K polymorphism is a variant discovered in the *PRNP* coding region (3). The PrP E219K homozygote leads to the loss of antigenic epitopes in PrP, which is defined as the KANNO-negative phenotype. KANNO-negative individuals may develop anti-KANNO alloantibodies upon pregnancy or transfusion (4, 5). Anti-KANNO can specifically recognize the KANNO antigen and wild-type (wt) PrP (2).

The PrP is the coding product of the *PRNP* gene in normal humans and animals (6, 7). It has been proposed to be involved in several cellular functions, such as circadian rhythm, calcium metabolism, tumor progression, myelin homeostasis, and immune modulation, but its importance still needs to be confirmed (8). Both normal and abnormal isoforms of PrP exist, and the latter is the main component leading to prion diseases (PrDs) (9, 10). Studies on the epidemiology (11–15) and spatial conformation (16–18) of PrP have suggested that the heterozygosity of the PrP E219K may influence the susceptibility to and phenotype of some forms of PrD. Nonetheless, the question of whether heterozygous inhibition can be used to resist prion infections remains unresolved.

There are no effective and noninvasive diagnostic or therapeutic strategies for human PrDs currently available in nonexperimental settings. The consideration of whether anti-KANNO antibodies specifically recognize prions provides new insight for intervention in PrDs. The PrP E219K polymorphism thus initially established an association between the KANNO blood group and human PrDs. Here, we review the KANNO blood group on a serological and molecular basis and discuss the moderation effect and possible mechanisms of the PrP E219K polymorphism on PrDs. We also explored the potential clinical application of the KANNO blood group in the diagnosis, prevention, and treatment of human PrD. We consider unanswered questions in the prion field that could contribute to methodological advances in our understanding of PrD.

## 2 The discovery of the KANNO blood group

### 2.1 Serological characteristics

The anti-KANNO antibody, named for the proband’s surname, was first discovered in the blood of a 49-year-old Japanese woman who underwent her first blood transfusion after hysterectomy in 1991 (4). The patient’s serum, containing anti-KANNO, reacted with 44 panel red blood cells (RBCs) lacking high-frequency antigens (HFAs) but did not aggregate with autologous RBCs; moreover, her RBCs were reactive with 48 other antisera against HFAs, which distinguishes this antibody from all known antibodies against HFAs (4, 5). Furthermore, an investigation of a total of 28 collected cases from Japan showed that anti-KANNO is an RBC alloantibody against nonself antigens and rarely occurs in the natural state but may develop in response to pregnancy or transfusion containing the KANNO antigen, where pregnancy may be the main stimulus (4, 5). Anti-KANNO antibodies are usually IgG, including IgG1 or the coexistence of IgG subsets, and react only in the indirect antiglobulin test (IAT) (5). Anti-KANNO produces weak reactions of similar strength from no dilution to higher titers in the IAT, which is characteristic of a high-titer, low-avidity (HTLA) antibody (19). However, anti-KANNO was distinguished from other HTLA antibodies through the examination of Kanno’s sera reacting with nonautologous RBCs that had been treated with enzymes, chemical reagents, complement sensitization, or serum neutralization (5). In addition, there were no reports of severe hemolytic transfusion reactions after transfusion with IAT-positive RBCs and no signs of hemolytic disease in the fetus or newborn caused by anti-KANNO in these patients, revealing limited clinical significance except for the slight impact on pretransfusion testing (4, 5). However, additional cases need to be tested to determine the diagnostic significance of anti-KANNO.

### 2.2 KANNO antigen and prion protein

To date, only one antigen molecule in the KANNO blood group system has been identified (2). The results of the monoclonal antibody (mAb)-specific immobilization of erythrocyte antigens (MAIEA) assay (20) indicate that the epitopes recognized by anti-KANNO and the known anti-human PrP mAb are located on the same membrane component but in distinct regions. This finding supports the notion that PrP is a cell membrane protein carrying the KANNO antigen. PrPs carry KANNO antigens but are not expressed only on the erythrocyte surface. PrPs are primarily active in the central and peripheral nervous systems and the lymphoreticular system. In particular, they are expressed at high levels by neurons in the brain and spinal cord and at comparatively low levels by neuroglial cells and numerous peripheral cell types, including peripheral blood cells. Most blood PrP is found in the plasma and platelet fractions (21, 22). PrP has been detected in all major human blood cell types, except for eosinophils (23), and humans express PrP at the highest levels on leukocytes and at medium levels on erythrocytes and platelets (24). One study estimated that approximately half of the PrP in blood cells is associated with RBCs and platelets, while less than 2% is associated with leukocytes, based on approximately 20 times and 2000 times more RBCs than platelets and leukocytes, respectively, in a given volume of blood (25). Human peripheral erythrocytes are reported to express as few as a median of 290 molecules but detectable amounts of PrP per cell, which makes them major contributors to the pool of PrP in blood due to the high number of RBCs (26). The results of flow cytometry indicate that lymphocytes and monocytes maintain PrP expression throughout their differentiation process, while PrP is downregulated upon differentiation into erythroid and granulocyte lineages (27, 28). PrP is a highly conserved membrane-bound protein across mammals, most of which are tethered by a glycosylphosphatidylinositol (GPI) anchor to the extracellular face of the plasma membrane (29). PrP is linked to the plasma membrane of peripheral blood leucocytes and platelets via a GPI anchor (30, 31). However, the trafficking of PrP in erythrocytes is not typical of GPI-linked proteins. PrP is reported to assemble on the cell surface of cultured human erythroblasts in tetraspanin-enriched microdomains and is then rapidly internalized into endosomal vesicles (28). The recycling process of PrP mediated by endosomes to the plasma membrane may also lead to the release of exosomes containing PrP molecules. The mechanism of PrP cycling in erythroblasts is similar to that in neuronal cells (32).

**Figure 1:**
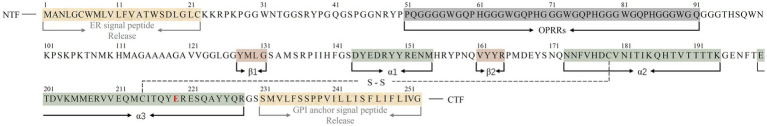
Sequence alignment of the full-length huPrP^C^ (23–230) monomer with three α-helices, two short β-sheets, and a single disulfide bond between Cys179 in the α2-helix and Cys214 in the α3-helix. Regions corresponding to α-helices, β-sheets and released amino acid residues in the protein monomer are indicated below the sequence (color bars covered). The C-terminal region of the α3-helix contains a polymorphism at codon 219, represented by a red E. A single disulfide bond is represented by S–S. The N-terminal fragment (NTF) and C-terminal fragment (CTF) are indicated in the sequence.

The human PrP (huPrP) has a canonical sequence of 253 amino acids^1^ and a molecular weight of approximately 33 ~ 35 kDa (6, 7). The precursor protein of huPrP consists of three characteristic regions: an N-terminal fragment (NTF) signal peptide with a nonapeptide region followed by 4 or 5 octapeptide repeat regions (OPRRs), a highly conserved hydrophobic region in the center of the protein, and a C-terminal fragment (CTF) hydrophobic region (6, 33). Nascent PrP is imported into the endoplasmic reticulum (ER) for partial folding and the formation of a disulfide bond between residues Cys179 and Cys214, which creates a loop of 36 amino acids containing both sites and further stabilizes the C-terminal structure (33). During translocation to the Golgi, PrP undergoes non, mono-, or di-glycosylation at two potential sites for N-linked glycosylation, namely, Asn181 and Asn197 (33). PrP was present mainly in its diglycosylated form on erythrocytes (26). After the release of 22 amino acid residues (ER signal peptide) at the NTF and 23 residues (GPI anchor signal peptide) at the CTF, the mature PrP, which consists of 208 amino acids and contains GPI anchors, is transported from the Golgi to the cell outer surface (33, 34). The final conformation of huPrP was primarily an N-terminal flexible tail with an irregular structure and an ordered C-terminal globular structure. The former contains a nonapeptide and OPRRs (residues 51 ~ 91), the latter contains two antiparallel β-sheets (residues 128 ~ 131 and 161 ~ 164), three α-helices (residues 144 ~ 154, 173 ~ 194, and 200 ~ 228), and a single Cys179-Cys214 disulfide bond linking the α2- and α3-helices (33, 35) (Figure 1).

PrP molecules are mainly classified into two forms: the host-encoded normal cellular isoform (PrP^C^) and the abnormal pathogenic “scrapie” isoform (PrP^Sc^). In general, PrP^C^ is monomeric, soluble in aqueous solution, and protease sensitive under physiological conditions (36). The physiological role of the PrP^C^ is not fully understood. Limited studies have demonstrated that the expression of PrP^C^ appears to be essential for the self-renewal, activation and developmental status of hematopoietic stem cells (37–39). Additionally, PrP^C^ expression on RBCs has been shown to correlate with their recovery and survival in circulation (40). A deficiency in PrP^C^ expression may lead to harm to the structure and function of RBCs, such as paroxysmal nocturnal hemoglobinuria (41) and acute anemia (42). PrP^C^ is also a component of plasma membrane microclusters and is involved in T lymphocyte signaling and activation (43). These findings suggest an important functional role of PrP^C^ in the hemopoietic system. In contrast, PrP^Sc^ displays insolubility against detergents, resistance to limited proteolysis, a high aggregation propensity, and a self-perpetuating characteristic relevant to PrDs (9, 10). Despite having obvious differences in their physicochemical and biological properties, the two PrP isoforms were confirmed by nucleotide sequencing to share the same DNA sequence (44). Analyses of PrP from various species using Fourier-transform infrared spectroscopy, mass spectrometry, and Edman sequencing revealed that the two isoforms had the same protein primary structure but distinct secondary structures characterized by significant changes in the proportions of α-helices and β-sheets; that is, α-helices were dominant in PrP^C^, while β-sheets with little, if any, α-helices were enriched in PrP^Sc^ (45–49). The formation of a misfolded, β-sheet-rich conformation has been proven to be the key mechanism of the pathogenicity of PrP^Sc^ (50). Understanding the structure of PrP^Sc^ is critical for elucidating its infectivity and toxicity. However, the high-resolution conformational characteristics of natural huPrP^Sc^ have not yet been characterized. The published molecular models suggest a diverse range of folds regarding the nature of the infectious conformer (51). Two primary near-atomic models were proposed for the core structure of highly infectious rodent brain-derived prions based on cryo-EM analysis and three-dimensional reconstruction experiments, as well as all other available experimental data (52). Among these, a parallel in-register intermolecular β-sheet-based architecture completely devoid of α-helices comprises stacked PrP monomers in the core of fibrils. Fibrils with single protofilaments coexist with twisted pairs of the same protofilaments (53, 54). Some fibrils show an asymmetric fibril cross-section without paired protofilaments (52). Each rung of the protofilament is formed by a single PrP monomer with an ordered core comprising PrP residues 94–225 (RML) or 95–227 (263 K), which folds and displays N-linked glycans and the GPI anchor of the C-terminus (52, 53, 55). In addition, these *ex vivo* prion strains share several structural motifs, including an N-terminal steric zipper and three β-arches, despite the disparate quantities of β-sheets identified in β-arches of different strains (55). A similar parallel, in-register architecture was observed in the fibril core derived from a huPrP mutation, which was associated with familial PrD. According to its near-atomic model, the fibril core maps to residues 108–141, where each subunit encompasses three relatively short β-strands (residues 109–112, 133–135, and 138–140) and the 113–125 region is rich in rigid turns (56). Another key model for the architecture of infectious mammalian prions is the four-rung β-solenoid structure (4RβS). This model is based on full-length PrP^Sc^ (57) and GPI-anchorless N-terminally truncated PrP 27–30 (58). The majority of fibrils observed in 4RβS were single-protofilament fibrils, accounting for nearly three-quarters of the total fibrils, and the remainder were two-protofilament fibrils that are twice as wide as single-protofilament fibrils (57). Some studies suggest that PrP^Sc^ is composed of a complex and heterogeneous ensemble of poorly defined conformations and quaternary arrangements (59, 60). This remains an important challenge for exploring its structural characteristics in the future.

### 2.3 Molecular basis: the *PRNP* codon 219 polymorphism

The KANNO antigen is encoded by the *PRNP* gene (NC_000020.11) (2), which maps to chromosome 20p13 (4,686,456 ~ 4,701,588) (61) and contains a 16-kb chromatin domain with two exons and one intron (7). The first exon acts as a transcription initiation site; the second exon contains the open reading frame that encodes huPrP (6, 7). The *PRNP* codon 219 polymorphism is one of more than 60 variants and polymorphisms discovered in the *PRNP* coding region (3). The presence of the *PRNP* codon 219 polymorphism is closely related to the seropositivity of the KANNO antigen. When the *PRNP* gene is affected by a homozygous missense polymorphism resulting in a G-to-A transition at the first position of codon 219 (c.655G > A, rs1800014) in the minor allele, a Glu to Lys substitution (E219K) in the amino acid sequence occurs, contributing to the loss of the antigenic epitopes of huPrP on the cell membrane (2). In contrast, *PRNPs* harboring 219E in the major alleles form antigenic epitopes of PrP (2). The available evidence suggests that anti-KANNO antibodies are capable of binding directly to *in vitro* cultured cells that possess wt PrPs but not to cells that have the 219 K/K homozygote or GPI anchor. Furthermore, sera obtained from KANNO-positive donors did not show any reactivity toward cells expressing wt PrPs. These findings, in conjunction with the results of previously mentioned serological tests, indicate that the KANNO-negative phenotype leads to the generation of anti-KANNO antibodies against Glu-type PrP (wt PrP) but not Lys-type PrP (2, 4, 5). However, there is currently no study that specifies which segment of PrP^C^ the anti-KANNO targets. Considering the location of this polymorphic locus, it is reasonable to assume that the antigenic determinants recognized by anti-KANNO are located within the C-terminal α3-helix region of PrP, including the 219E site (Figure 1). It remains uncertain whether the sequence epitopes of the KANNO antigen are either linear or discontinuous. Notably, several established techniques may be available for the characterization of KANNO epitopes, including mutagenic library sorting, peptide arrays, alanine scanning, and co-crystallization (62). In addition, the 219 K allele acts as a recessive trait. However, family studies of four KANNO-negative patients showed that all the immediate family members, including the proband, observed in independently recruited families had no close relatedness in their genotyped samples (2). The genetic characterization of the KANNO blood group requires further clarification in additional cases.

The naturally occurring E219K polymorphism of *PRNPs* in humans was first discovered in the genomes of Japanese patients with schizophrenia and was found to carry 6% of the 219 K allele frequency in the Japanese general population (63). Worldwide, the distribution of allele frequencies of this polymorphism in the general population was found to show west–east gradient-raised patterns (2, 64–68), as shown in Table 1. Furthermore, E/K heterozygosity at codon 219 was reported only in East Asian populations (Table 2). The general population in Japan has a slightly greater genotype frequency of 219E/K than that in South Korea and China (11, 63, 69–75). In the healthy Chinese population, the genotype frequency of the E219K polymorphism differed according to ethnicity and region (71). Notably, homozygous 219 K/K *PRNPs* with a low but appreciable genotype frequency (approximately 0.3 ~ 0.9%) were discovered only in Japanese and South Korean native populations (2, 73, 75), as well as Punjabi from Lahore, Pakistan and Gujarati Indians from Houston, Texas, based on the 1,000 Genomes database and the gnomAD database (2). This finding also represents the distribution of the KANNO-negative phenotype in the general population.

**Table 1:** Allele frequencies of the PrP E219K polymorphism in global populations.

Population	Allele frequency (%)		References
	E	K	
Asian	97.4	2.6	(64)
East Asian	90.6 ~ 96.5	3.5 ~ 9.4	(2, 65)
Central-East Asian	97.46	2.54	(66)
South Asian	87.2 ~ 99.1	0.9 ~ 12.8	(2, 65, 66)
Pacific	88.2 ~ 90.6	9.4 ~ 11.8	(65, 66)
Melanesian	81 ~ 90	10 ~ 19	(64, 66)
Oceanian	94.4	5.6	(64)
Middle East/African	99.4	0.6	(64)
African	100	0	(65)
Subsaharan African	100	0	(64)
Latin American	100	0	(64)
South American	100	0	(65)
European	100	0	(64)

**Table 2:** Genotype frequencies of the PrP E219K polymorphism in the general population.

Population	Genotype frequency (%)			References
	E/E	E/K	K/K	
Japanese	85.6 ~ 88.0	12.0 ~ 14.4	0 ~ 0.7	(2, 63, 69)
South Korean	90.32 ~ 92.06	7.82 ~ 8.76	0 ~ 0.92	(2, 11, 70~^73^)
Chinese	84.2 ~ 97.8	2.2 ~ 15.8	0	(70, 71)
Punjabi from Lahore	NA^a^	NA^a^	1.04	(2)
Gujarati Indians from Houston	NA^a^	NA^a^	1.94	(2)
Serbian	100	0	0	(67)
Italian	100	0	0	(68)

^a^Not available.

## 3 The impact of the PrP E219K polymorphism on prion diseases

### 3.1 Genetic susceptibility of E219K to different forms of human PrD

*PRNP* was established as the only causative gene for PrDs (76), and polymorphisms in human *PRNPs* were found to modify susceptibility to and the phenotype of PrDs. E219K, which has an undefined pathogenic nature, is by far the most important human *PRNP* polymorphism except M129V, that is, a Met to Val substitution at codon 129 (77, 78). The nature of M129V and E219K is complicated, and both are suggested to play neutral or protective roles but may also be risk- or disease-modifying factors for different forms of PrD. Human PrDs can be categorized into three forms according to their etiology: sporadic, genetic, and acquired (3). PrP E219K may influence susceptibility to sporadic Creutzfeldt–Jakob disease (sCJD), which accounts for more than 90% of all cases of sporadic PrDs. Cohort studies from East Asian countries reported that patients with dementia of non-CJD origin had similar genotypes and allele and/or haplotype frequencies of E219K to those of the general population (12, 69, 70, 73, 74, 79), whereas the K allele and E/K heterozygosity at codon 219 appeared in general controls (4 ~ 6% and 8 ~ 12%, respectively) but were not found or had a very low frequency (0% and 0 ~ 0.53%, respectively) of sCJD patients in these countries, revealing a significant discrepancy (11–15). Limited studies from European countries have shown that only the wt *PRNP* sequence, not the K allele, is present at codon 219 in both sCJD patients and in the general population (68, 80, 81). There are no reports of PrP E219K in other regional populations. It is not feasible to establish a correlation between the E219K polymorphism and sCJD in these countries or regions lacking the K allele. Therefore, the protective effect of E219K heterozygosity against sCJD has been limited to East Asian populations to date.

Data from Japanese CJD surveillance showed that the frequencies of the K allele and the heterozygous genotype at codon 219 of *PRNPs* in genetic CJD (gCJD) patients (0 ~ 1.3% and 0 ~ 2.2%) were significantly lower than those in the general controls (14). Four cases of gCJD in China (82, 83) and 50 individuals from four families affected by gCJD in Chile (84) were also detected with only 219E/E homozygosity. The codon 219 polymorphism, harboring Methionine homozygosity at codon 129, appears to have a protective effect against both gCJD and Gerstmann-Straussler-Scheinker disease (GSS), according to a Japanese population dataset (78). E219K also affects the pathogenicity of gPrD-related mutations. There is potential protection of E219K against gCJD-V180I onset based on tested cases with V180I carrying the 219E/E homozygote (15). The coexistence of E219K with the E200K mutant in the same allele prolonged the duration of mild clinical symptoms of gCJD, which differs from the clinical phenotype of typical E200K gCJD (85, 86). A genotype pattern with 219 K present in the P102L mutant allele also seems to be an important factor alleviating neurological and pathological symptoms in Japanese patients with GSS compared with patients carrying P102L and E219K in different alleles (63, 87, 88). Similarly, one of 12 Chinese patients with GSS-P102L was heterozygous for E219K, and this patient appeared to have less severe symptoms than other patients (89). However, in contrast to the potential heterozygous inhibition of E219K in the clinical course of GSS, the presence of 219 K within the P102L mutant allele may permit the formation of abnormal PrP, indicating a risk- or disease-modifying factor (90). Moreover, reports on *PRNP* 219 K/K homozygotes are also extremely limited. Among Japanese gCJD patients, 219 K/K homozygotes had a genotype frequency of 0.52% (14), similar to that in the general population of Japan. Only one case report described a Japanese patient with probable familial CJD (fCJD) carrying an OPRR insertion and homozygous codon 219 K (91). The latter may be more prone to modification of the clinical course and pathologic features of disease by unknown mechanisms and may not offer sufficient protection against gCJD (91), although there is insufficient information to demonstrate that 219 K/K promotes gCJD. In addition, the E219K polymorphism may not influence susceptibility to fCJD/GSS in Caucasians because of the absence of the K allele in both patients and healthy people (68, 92).

Reports about the PrP E219K polymorphism in acquired PrD have rarely been published. Dura mater graft-associated CJD (dCJD), which is of iatrogenic origin, has a relatively high incidence among patients with acquired PrD. The frequencies of the K allele and 219E/K heterozygosity of *PRNPs* in dCJD (1.95 and 3.9%, respectively) were not significantly different from those in the Japanese general population (14). Two Japanese cases indicated that the codon 219 K allele may be associated with mild clinicopathological features in dCJD, such as a long incubation period, atypical periodic sharp-wave complexes (PSWCs), and nonplaque type (93, 94). However, other scattered cases have shown that 219E/K heterozygosity may not influence the disease processes of dCJD and may not render humans sufficiently resistant to direct invasion of infectious prions into the brain from dura mater grafts (12). In addition, few systematic studies of the E219K polymorphism have been reported to date for another subtype of acquired PrD, variant CJD (vCJD). There is a relatively low incidence of vCJD in Asia. During 10 years of surveillance, only one patient with vCJD was reported in Japan, and the patient did not carry the *PRNP* 219 K allele (14). Although the incidence of vCJD in Caucasians is significantly greater than that in East Asians (95), Caucasians have rarely been reported to carry the PrP E219K. In a UK study, a total of two vCJD patients harboring E219K heterozygosity were found to be of non-Caucasian origin and had no clinicopathological differences from classical vCJD patients (80). Two other UK cases suggested that the heterozygous genotype at codon 219 was not resistant to vCJD and might even increase susceptibility and risk (96). It remains challenging to delineate the correlation between E219K and the acquired PrD based on the extremely limited data at present.

Overall, the PrP E219K polymorphism affects susceptibility to PrDs and the clinical course and pathological features of patients, which is restricted by etiology- and race-related patterns. However, some studies have examined only a limited number of patients, especially those with genetic and acquired PrDs, and may be considered too preliminary. The findings regarding the role of PrP E219K in modifying the genetic susceptibility of humans to PrD necessitate verification in studies involving larger sample sizes or in regional populations other than Asia.

### 3.2 Conformational properties and effects of E219K on prion infection models

Prion conversion is regarded as a method of protein self-replication in which PrP^Sc^ acts as a template that converts PrP^C^, as a substrate, into a nascent PrP^Sc^ molecule (3, 76). Codon 219 of huPrP is at the C-terminal end of the α3-helix (Figure 1), which is part of the globular domain, the core region of the PrP conformation (33, 35). The E219K polymorphism affects the flexibility of the core structures based on models constructed by multiple methods (97). Further examination revealed elongation of the E219K-associated helix and increases in the overall flexibility and stabilizing interactions of the hydrophobic core (98). The structural and kinetic information from Markov state model analyses also suggested that the heightened stability of the E219K mutant was associated with an increase in the level of native contacts and robust salt bridges and a decrease in random motions (99). In E219K huPrP, strong hydrophobic contacts are present between residues from the β2-α2 loop and the C-terminal segment of the α3-helix (17). The E219K polymorphism reduced the backbone flexibility and/or conformational exchange processes of huPrP, indicating a tendency toward a rigid β2-α2 loop conformation that has been shown to be resistant to prion infections and to not cause spontaneous PrDs to develop (100–102). However, another study involving molecular dynamics simulations demonstrated that increased flexibility in the β2-α2 loop can be observed in E219K huPrP compared with wt huPrP (103). The disagreement between the various modeling solutions indicates the limitations of each method. Moreover, 219 K huPrPs in homozygous or hemizygous forms are PrP^Sc^ conversion competent and can pile up into amyloid fibrils in knock-in mouse models expressing chimeric human/mouse PrP (16). The structure–function inconsistency observed in 219 K huPrP may be explained to some extent by a variable transmission barrier that can be assessed by the degree of permitted sequence and conformational overlap of PrP between the pathogen and the host (96), reflecting the importance of homology (104). The β2-α2 loop within PrP varies substantially between species, and similar loop structures correlate with efficient conversion, whereas dissimilar loops correlate with strong transmission barriers (100). Additionally, solution nuclear magnetic resonance (NMR) studies suggested that the E219K substitution caused severe perturbation of the surface electrostatic potential and subtle local structural rearrangements within the key epitopes of PrP molecule conversion (17). The E219K PrP exhibited large areas of positive charge around the site of substitution, extending to regions of the β2-α2 loop and α3-helix, whereas the corresponding regions of the wt protein were negatively charged or neutral (17, 18). Differences in the distribution of charges and incompatible structures and dynamics in the core regions of PrP may promote intermolecular interference between the 219E and 219 K PrPs, thus sequestering each other in the early stages of the fibrillization and stacking process, referred to as heterozygous inhibition (16, 18).

The corresponding E219K huPrP homolog, a Gln to Lys substitution at codon 218 (Q218K) of mouse PrP (moPrP), has been shown to inhibit both the conversion and stacking of PrP^Sc^ via the conversion-incompetent 218 K PrP, referred to as dominant-negative inhibition (105, 106). Combined mutagenesis and structural studies suggested that the β2-α2 loop and α3-helix together form a discontinuous epitope on the protein surface that has been proposed to constitute the binding site for “protein X” (107), a putative yet unidentified chaperone-like molecule that was proposed to exhibit greater affinity for homologous PrP^C^ by species specificity to confer more efficient binding to PrP^Sc^ (108). Consistently, studies have demonstrated that inhibition of prion propagation *in vivo* may occur through the binding of drugs to residues within the C-terminal α3-helix of human recombinant PrP (rPrP), which is situated near the “protein X” epitope (109). Within this epitope, 218 K moPrP was demonstrated to prevent pathological conversion of its own and even wt PrP^C^ by binding tightly to protein X and segregating it from the PrP^Sc^ replication process, acting as a dominant-negative inhibitor in *in vitro* or *in vivo* prion models (105–107). Furthermore, kringle and other domains have been identified as potential epitopes for the specific, high-affinity binding of 218 K moPrP to protein X and the binding of other PrPs to it (110). However, it is frustrating that the role of any protein in PrP conversion has not been well established, despite many proteins being known to bind to PrP^C^. Moreover, in cell-free conversion reactions in the absence of a PrP^Sc^ template or an external cofactor, 218 K mouse rPrP also showed dominant-negative inhibition by reducing the formation of amyloid fibrils and the yield of polymerization of wt rPrP (111). This finding indicates that “protein X” is not essential for mediating dominant inhibition of prion propagation. In addition, in serial protein misfolding cyclic amplification reactions, the 219 K hamster rPrP molecule, as a substrate, readily converts into self-propagating PrP^Sc^ and inhibits wt rPrP conversion but not vice versa (112), displaying trans-dominant inhibitory activity. This effect can be explained by the fact that the 219 K mutant has a greater affinity than wt PrP^C^ for binding to the nascent seeding site on the growing PrP^Sc^ polymer (112).

Despite the observation that 219 K huPrP and its homologs can inhibit the conversion of wt PrP^C^ to PrP^Sc^, the underlying mechanisms, such as rigid conformations, incompatible structures or competitive binding, are different. These mechanisms require further clarification, but it can be inferred that they are closely associated with the conformational properties of PrP strains harboring these polymorphisms from different species. Notably, based on the heterozygous inhibition of PrP E219K, several low-molecular-weight compounds have been designed to develop therapeutic strategies to inhibit prion infections (113). Unfortunately, none of these compounds has yet been successfully translated into effective therapies for PrDs in nonexperimental settings. Lentiviral gene transfer of E219K was shown to abolish the conversion and replication of endogenous wt PrP^C^ into PrP^Sc^ *in vitro* only, although this method has been shown to transduce cells in brain tissues *in vivo* (114). Therefore, the impacts and mechanisms of the PrP E219K polymorphism on human PrDs still need to be clarified by further improving the fine structural models of the essential entities of PrD (fibril PrP^Sc^) and 219 K PrP molecules derived from humans and analyzing their characteristics (115).

## 4 Potential clinical applications of the KANNO blood group

**Figure 2:**
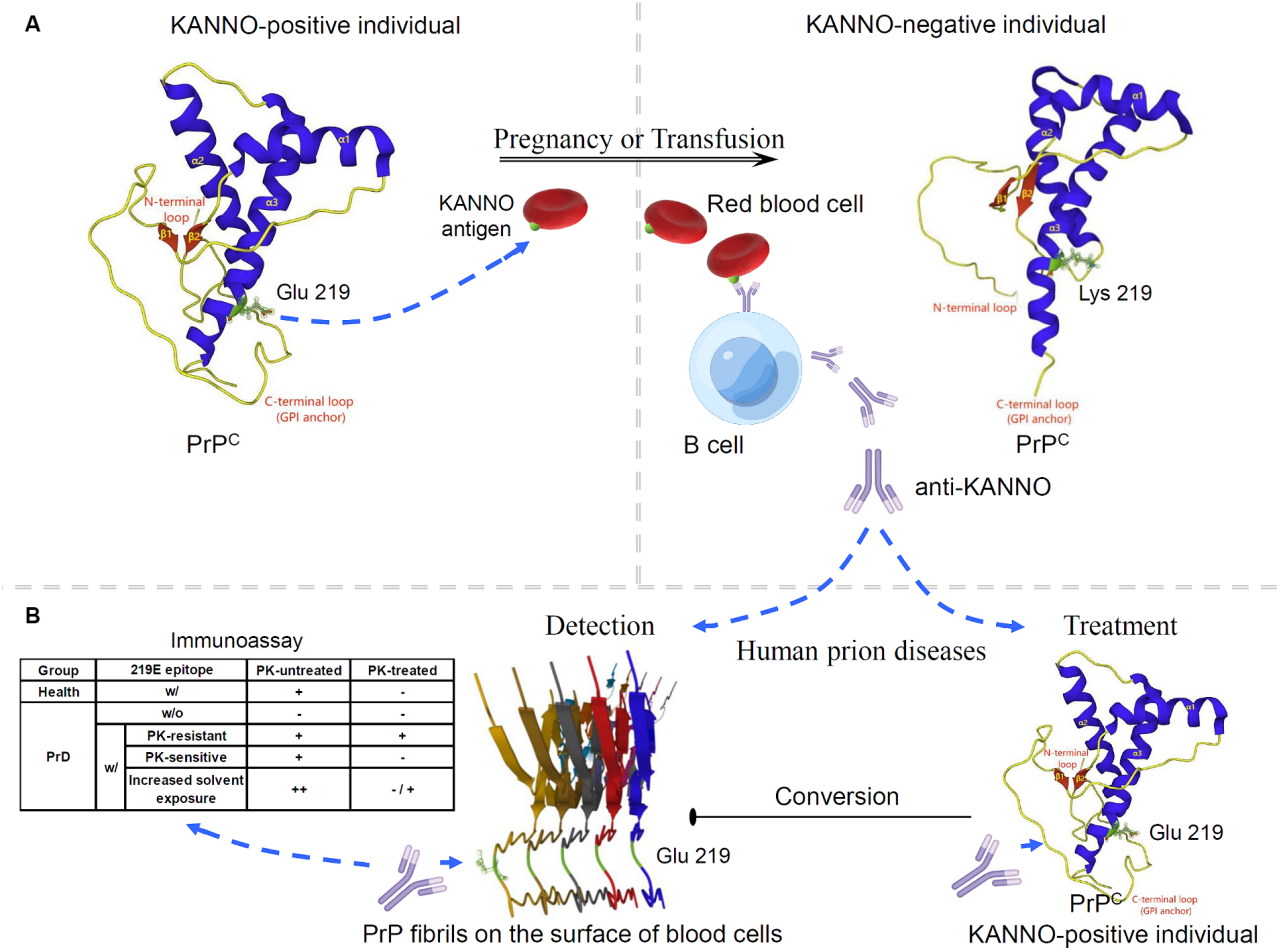
Potential application of the KANNO blood group in interventions for human prion diseases. **(A)** The anti-KANNO alloantibody is produced in patients with the KANNO-negative phenotype and may develop in response to pregnancy or transfusion with KANNO antigens. **(B)** The degree of anti-KANNO recognition of cell-surface PrP on peripheral blood cells from PrD patients is determined by an immunoassay and compared to that on peripheral blood cells from healthy individuals. The table lists several theoretically plausible outcomes regarding the possibility of anti-KANNO binding to PrP^Sc^ (w/: with; w/o: without). Additionally, anti-KANNO may bind selectively to PrP^C^ to inhibit its conversion into PrP^Sc^ *in vivo*. The solution NMR spectra of 219E huPrP (90 ~ 231; PDB ID: 2LSB) and 219 K huPrP (90 ~ 231; PDB ID: 2LFT) reveal an N-terminal flexible tail with an irregular structure (yellow) and an ordered C-terminal globular structure. The latter contains three α-helices (blue) and two antiparallel β-sheets (red). Codon 219 of huPrP is in the C-terminal secondary structure of the α3-helix (green). Glu-type PrP expresses at least one E version at codon 219 in the *PRNP* alleles, and Lys-type PrP is homozygous for E219K. The cryo-EM structure of human rPrP fibrils shows enrichment of β-sheets in the C-terminal domain (170 ~ 231; PDB ID: 6LNI).

Given the limited progress in the use of E219K heterozygous inhibition for the treatment of PrDs, we attempted to discuss the application of new biological functions of this polymorphism in PrDs. Currently, no studies have evaluated the effect of KANNO on human disease-associated PrP. However, the discovery of the KANNO blood group based on the genetic association between the KANNO antigen and the huPrP molecule can provide new insight for prion intervention. The KANNO antigen carrying the 219E allele on RBCs may immunize KANNO-negative individuals harboring the 219 K/K homozygote to generate anti-KANNO alloantibodies that are specifically reactive to the native conformation of wt huPrP (2, 4, 5) (Figure 2A). The structural domain constructed by the huPrP codon 219E is immunogenic in KANNO-negative individuals, although the immune response caused by anti-KANNO is very slight, with no clinical symptoms (4, 5). This phenomenon provides an anti-KANNO therapeutic strategy with potential application for inhibiting the conversion to disease-related PrP^Sc^ by targeting wt PrP^C^ during prion infection (Figure 2B). Experimental studies on therapeutic strategies for PrD demonstrated that mAbs reacting to at least four domains of moPrP seem to effectively clear prion infection and abrogate nascent PrP^Sc^ formation in a cell-free or cell culture system, including OPRR, spanning residues 90–110, α1-helix region residues 145–160, and extreme C-terminal residues 210–220 (116–119). Furthermore, it has been shown that peptides and antibodies corresponding to the C-terminus prevent the formation of PrP amyloid fibrils *in vivo* (120). We speculate that anti-KANNO may inhibit PrP fibril stacking by targeting 219E between two β-sheet regions within the β-sheet conversion core of rPrP aggregates (121, 122). In addition, mAbs confer anti-prion potency *in vivo*, inhibiting prion transport from peripheral sites to the central nervous system (CNS), prolonging the incubation period and delaying disease onset (123–125), but only after peripheral infection with prions, not after intracerebral infection (116). Even when the mAb at a much higher dosage were first administered at the point of near maximal accumulation of peripheral PrP^Sc^, it showed markedly inhibitory effects on peripheral PrP^Sc^ levels and completely prevent disease onset (125), but has not been found to be effective late in the incubation period or closer to the clinically symptomatic stages of prion infection, and also has not showed effects on disease progression or mortality (126, 127). Limitation of treatment time-window is probably due to relatively large molecules of anti-PrP with the blood–brain barrier (BBB) impermeability (128, 129). To achieve effective CNS delivery of full-length IgG antibodies, such as anti-KANNO, transport across the BBB is a major challenge. One approach to improving delivery efficiency is to design an anti-KANNO Fc domain to target an epitope on the extracellular domain of the endogenous BBB receptor (130, 131). Furthermore, despite their high invasiveness, BBB avoidance strategies, including the intrathecal or intracerebroventricular delivery of anti-KANNO into the cerebrospinal fluid circulation (132), may effectively deliver anti-KANNO to the brain extracellular space. Considering these perspectives, this approach will show the advantages of anti-KANNO, a genuine human antibody, in delaying or inhibiting the progression of PrDs, at least as prophylaxis in the presymptomatic phase. The efficacy of anti-KANNO antibodies is anticipated to be determined by the outcome of future clinical trials. A comparison of anti-KANNO with a mouse mAb raised against huPrP known as PRN100 (an IgG4κ isotype), which was successfully developed as a clinical candidate designed to bind and stabilize huPrP^C^, was recommended, and this study was conducted in a first-in-human trial of patients with CJD (133, 134).

Notably, peripheral blood is considered a possible reservoir of prion infectivity in PrP^Sc^-affected individuals. It has been demonstrated that PrD can be transmitted through transfusion of whole blood or buffy coats obtained during the presymptomatic and symptomatic phases of infection from either natural or experimental PrP^Sc^-infected mammalian species (135–137). Furthermore, native PrP^Sc^ was detected in fresh whole blood from vCJD patients and scrapie-infected animals by using specific antibodies that selectively recognize PrP^Sc^ in distinct immunoassays (138–140). Similarly, PrP^Sc^ was found to be a key membrane fraction isolated from whole blood in naturally affected scrapie animals (141). The PrP molecules on the surface of peripheral blood mononuclear cells from scrapie-infected animals also showed structural changes that may be relevant to PrD, as demonstrated by alterations in the recognition of the anti-PrP mAb (142). Moreover, hematogenous transmission of infection between different peripheral lymphatic reticular sites is a recognized characteristic of the preclinical stage of PrD in experimental rodent infection following nonneural inoculation (143–145). However, investigations conducted on both exogenous and endogenous prions affecting rodent and human blood have shown that infectivity in RBC preparations is not intrinsic to the RBCs themselves but rather occurs within the suspending medium, where it can be easily removed by filtration (146–149). The opposing perspective posits that while there is no direct evidence that PrP^Sc^ is anchored on RBCs, according to the “protein-only” theory, PrP^Sc^ serves as a template for converting the host’s native PrP^C^ by physical interaction. Therefore, any cell expressing PrP^C^ could theoretically serve as a receptor, a site for propagating infectivity, or both (25, 150). The conversion of PrP^C^ to PrP^Sc^ is believed to occur at the cell surface or during the endocytosis cycle (151). RBCs and platelets may bind to PrP^Sc^ introduced through a peripheral infection (152). Taken together, these findings suggest that peripheral blood cells, particularly mononuclear cells, may be suitable targets for early screening, diagnosis or monitoring of PrP-related diseases. Furthermore, we also aimed to determine whether anti-KANNO antibodies may be effective in targeting disease-associated PrP present on the surface of blood cells. Briefly, plasma/sera containing natural anti-KANNO are collected from KANNO-negative individuals who have received transfusion or are pregnant, and peripheral blood cells are collected from KANNO-positive patients in the suspected presymptomatic or symptomatic phase of PrDs. The degree of anti-KANNO recognition of cell-surface PrP is then determined by performing an immunoassay on peripheral blood cells from PrD patients and compared to those from healthy individuals, with or without the use of proteinase K (PK) digestion as a criterion (Figure 2B). Antibody affinity and efficacy are epitope-dependent. Notably, whether anti-KANNO recognizes PrP^Sc^ depends on whether the epitope recognized by anti-KANNO is altered upon the conversion of PrP^C^ to PrP^Sc^. It is crucial to address the following questions for this proof-of-concept study: first, whether pathogenic PrP on the surface of peripheral blood cells from prion-infected patients shares epitopes mapped to the Glu residue at position 219 of huPrP with PrP^C^; second, whether the abnormal protein contains a C-terminal PK-resistant core with the aforementioned epitopes; and third, whether the accessibility of the C-terminal region of the α3-helix increases due to increased solvent exposure in PrP^Sc^ on peripheral blood cells from PrD patients, leading to an increase in anti-KANNO recognition of these epitopes.

Thorough characterization of the dynamic conformational landscapes of huPrP^Sc^ will be helpful for identifying physiologically relevant and druggable transitions of anti-KANNO. The original insight into the clinical application of the KANNO blood group highlights unique features that make anti-KANNO targeting PrP an attractive and rational approach for developing therapeutic reagents for PrDs and alternative diagnostic strategies. Anti-KANNO may also be used in blood-detection procedures in humans, which may improve the safety of blood products and reduce the risk of further spread of PrDs. The practical application of the KANNO blood group must be tested experimentally before any practical conclusions can be drawn.

## 5 Conclusion

Our current understanding indicates that there are two important clinically significant effects of the PrP E219K polymorphism. First, this polymorphism is a genetic factor that modifies susceptibility to human PrDs and the phenotype of human PrDs, especially sCJD, in Asia. Although E219K heterozygous inhibition is reflected in genetic susceptibility to human PrDs and changes in the conformational properties of PrP, methods for preventing or treating prion infection based on this mechanism have yet to be developed. Second, it is the molecular basis of the KANNO blood group. Since the KANNO blood group was confirmed, it is considered to have a minimal impact on the safety of blood transfusions and limited clinical significance. However, based on the conformational properties of the E219K polymorphism in the *PRNP*, the determinant gene of the KANNO blood group, we suggest that this blood group has potential new applications in PrD intervention.

The *PRNP* gene encodes both PrP^C^ and PrP^Sc^. Regardless of whether they share epitopes constructed by the Glu residue of codon 219, it is possible to distinguish alterations in anti-KANNO recognition of cell-surface PrP on peripheral blood cells from PrD patients. Anti-KANNO may serve as a noninvasive auxiliary immunoassay tool for the diagnosis of certain human PrDs. Anti-KANNO may also play an important role in inhibiting the conversion of PrP^Sc^ by directly targeting PrP^C^. Future studies should focus on improving the delivery of anti-KANNO to the CNS and assessing its safety in the brain, as well as investigating its potential roles in PrD diagnosis and treatment. This *bona fide* human antibody is expected to prevent or treat human PrD at least at an early stage.

## Author contributions

S-SW: Funding acquisition, Investigation, Writing – original draft, Writing – review & editing. Z-LM: Writing – original draft. Y-WZ: Writing – original draft. Y-SY: Writing – original draft. L-BL: Investigation, Writing – review & editing.

## Footnotes

^1^ https://www.ncbi.nlm.nih.gov/nuccore/NG_009087.1

## References

[1] Gassner C Castilho L Chen Q Clausen FB Denomme GA Flegel WA. International Society of Blood Transfusion Working Party on red cell Immunogenetics and blood group terminology report of Basel and three virtual business meetings: update on blood group systems. Vox Sang. (2022) 117:1332–44. doi: 10.1111/vox.13361, PMID: 36121188 PMC10680040

[2] Omae Y Ito S Takeuchi M Isa K Ogasawara K Kawabata K. Integrative genome analysis identified the KANNO blood group antigen as prion protein. Transfusion. (2019) 59:2429–35. doi: 10.1111/trf.15319, PMID: 31020675

[3] Kim MO Takada LT Wong K Forner SA Geschwind MD. Genetic PrP prion diseases. Cold Spring Harb Perspect Biol. (2018) 10:a033134. doi: 10.1101/cshperspect.a033134, PMID: 28778873 PMC5932589

[4] Kawabata K Yasuda H Tsuchida H Ito S Kikuchi M Tsuneyama H. Serologic reactivity and clinical significance of the high frequency antigen KANNO and its antibody. Japanese J Transfusion Cell Ther. (2011) 57:478–83. doi: 10.3925/jjtc.57.478

[5] Kawabata K Uchikawa M Ohto H Yasuda H Tsuneyama H Tsuchida H. Anti-KANNO: a novel alloantibody against a red cell antigen of high frequency. Transfus Med Rev. (2014) 28:23–8. doi: 10.1016/j.tmrv.2013.12.001, PMID: 24485899

[6] Bernardi L Bruni AC. Mutations in prion protein gene: pathogenic mechanisms in C-terminal vs. N-terminal domain, a review. Int J Mol Sci. (2019) 20:3606. doi: 10.3390/ijms20143606, PMID: 31340582 PMC6678283

[7] Puckett C Concannon P Casey C Hood L. Genomic structure of the human prion protein gene. Am J Hum Genet. (1991) 49:320–9. PMID: 1678248 PMC1683278

[8] Kovač V Čurin ŠV. Prion protein: the molecule of many forms and faces. Int J Mol Sci. (2022) 23:1232. doi: 10.3390/ijms23031232, PMID: 35163156 PMC8835406

[9] Rossetti G Giachin G Legname G Carloni P. Structural facets of disease-linked human prion protein mutants: a molecular dynamic study. Proteins. (2010) 78:3270–80. doi: 10.1002/prot.22834, PMID: 20806222

[10] Prusiner SB. Molecular biology of prion diseases. Science. (1991) 252:1515–22. doi: 10.1126/science.1675487 1675487

[11] Jeong BH Lee KH Kim NH Jin JK Kim JI Carp RI. Association of sporadic Creutzfeldt-Jakob disease with homozygous genotypes at PRNP codons 129 and 219 in the Korean population. Neurogenetics. (2005) 6:229–32. doi: 10.1007/s10048-005-0016-y, PMID: 16217673

[12] Shibuya S Higuchi J Shin RW Tateishi J Kitamoto T. Codon 219 Lys allele of PRNP is not found in sporadic Creutzfeldt-Jakob disease. Ann Neurol. (1998) 43:826–8. doi: 10.1002/ana.410430618, PMID: 9629853

[13] Shibuya S Higuchi J Shin RW Tateishi J Kitamoto T. Protective prion protein polymorphisms against sporadic Creutzfeldt-Jakob disease. Lancet. (1998) 351:419. doi: 10.1016/s0140-6736(05)78358-6 9482303

[14] Nozaki I Hamaguchi T Sanjo N Noguchi-Shinohara M Sakai K Nakamura Y. Prospective 10-year surveillance of human prion diseases in Japan. Brain. (2010) 133:3043–57. doi: 10.1093/brain/awq216 20855418

[15] Qina T Sanjo N Hizume M Higuma M Tomita M Atarashi R. Clinical features of genetic Creutzfeldt-Jakob disease with V180I mutation in the prion protein gene. BMJ Open. (2014) 4:e004968. doi: 10.1136/bmjopen-2014-004968, PMID: 24838726 PMC4025468

[16] Hizume M Kobayashi A Teruya K Ohashi H Ironside JW Mohri S. Human prion protein (PrP) 219K is converted to PrPSc but shows heterozygous inhibition in variant Creutzfeldt-Jakob disease infection. J Biol Chem. (2009) 284:3603–9. doi: 10.1074/jbc.M809254200, PMID: 19074151

[17] Biljan I Ilc G Giachin G Legname G Plavec J. NMR structural studies of human cellular prion proteins. Curr Top Med Chem. (2013) 13:2407–18. doi: 10.2174/15680266113136660169 24059340

[18] Biljan I Giachin G Ilc G Zhukov I Plavec J Legname G. Structural basis for the protective effect of the human prion protein carrying the dominant-negative E219K polymorphism. Biochem J. (2012) 446:243–51. doi: 10.1042/bj20111940, PMID: 22676969

[19] Rolih SD. High-titer, low-avidity (HTLA) antibodies and antigens: a review. Transfus Med Rev. (1989) 3:128–39. doi: 10.1016/s0887-7963(89)70074-2, PMID: 2520549

[20] Petty AC. Monoclonal antibody-specific immobilisation of erythrocyte antigens (MAIEA). A new technique to selectively determine antigenic sites on red cell membranes. J Immunol Methods. (1993) 161:91–5. doi: 10.1016/0022-1759(93)90200-q, PMID: 8486932

[21] MacGregor I Hope J Barnard G Kirby L Drummond O Pepper D. Application of a time-resolved fluoroimmunoassay for the analysis of normal prion protein in human blood and its components. Vox Sang. (1999) 77:88–96. doi: 10.1046/j.1423-0410.1999.7720088.x, PMID: 10516553

[22] Bessos H Drummond O Prowse C Turner M MacGregor I. The release of prion protein from platelets during storage of apheresis platelets. Transfusion. (2001) 41:61–6. doi: 10.1046/j.1537-2995.2001.41010061.x, PMID: 11161247

[23] Barclay GR Houston EF Halliday SI Farquhar CF Turner ML. Comparative analysis of normal prion protein expression on human, rodent, and ruminant blood cells by using a panel of prion antibodies. Transfusion. (2002) 42:517–26. doi: 10.1046/j.1537-2995.2002.00095.x, PMID: 12084159

[24] Holada K Simak J Brown P Vostal JG. Divergent expression of cellular prion protein on blood cells of human and nonhuman primates. Transfusion. (2007) 47:2223–32. doi: 10.1111/j.1537-2995.2007.01451.x, PMID: 17714417

[25] Vostal JG Holada K Simak J. Expression of cellular prion protein on blood cells: potential functions in cell physiology and pathophysiology of transmissible spongiform encephalopathy diseases. Transfus Med Rev. (2001) 15:268–81. doi: 10.1053/tmrv.2001.26957, PMID: 11668434

[26] Panigaj M Brouckova A Glierova H Dvorakova E Simak J Vostal JG. Underestimation of the expression of cellular prion protein on human red blood cells. Transfusion. (2011) 51:1012–21. doi: 10.1111/j.1537-2995.2010.02924.x 21058954

[27] Dodelet VC Cashman NR. Prion protein expression in human leukocyte differentiation. Blood. (1998) 91:1556–61. doi: 10.1182/blood.V91.5.1556, PMID: 9473220

[28] Griffiths RE Heesom KJ Anstee DJ. Normal prion protein trafficking in cultured human erythroblasts. Blood. (2007) 110:4518–25. doi: 10.1182/blood-2007-04-085183, PMID: 17827389

[29] Stahl N Borchelt DR Hsiao K Prusiner SB. Scrapie prion protein contains a phosphatidylinositol glycolipid. Cell. (1987) 51:229–40. doi: 10.1016/0092-8674(87)90150-4, PMID: 2444340

[30] Barclay GR Hope J Birkett CR Turner ML. Distribution of cell-associated prion protein in normal adult blood determined by flow cytometry. Br J Haematol. (1999) 107:804–14. doi: 10.1046/j.1365-2141.1999.01789.x, PMID: 10606888

[31] Dürig J Giese A Schmücker U Kretzschmar HA Dührsen U. Decreased prion protein expression in human peripheral blood leucocytes from patients with paroxysmal nocturnal haemoglobinuria. Br J Haematol. (2001) 112:658–62. doi: 10.1046/j.1365-2141.2001.02602.x 11260069

[32] Sunyach C Jen A Deng J Fitzgerald KT Frobert Y Grassi J. The mechanism of internalization of glycosylphosphatidylinositol-anchored prion protein. EMBO J. (2003) 22:3591–601. doi: 10.1093/emboj/cdg344, PMID: 12853474 PMC165614

[33] Acevedo-Morantes CY Wille H. The structure of human prions: from biology to structural models-considerations and pitfalls. Viruses. (2014) 6:3875–92. doi: 10.3390/v6103875, PMID: 25333467 PMC4213568

[34] Heller U Winklhofer KF Heske J Reintjes A Tatzelt J. Post-translational import of the prion protein into the endoplasmic reticulum interferes with cell viability: a critical role for the putative transmembrane domain. J Biol Chem. (2003) 278:36139–47. doi: 10.1074/jbc.M304002200, PMID: 12853456

[35] Zahn R Liu A Lührs T Riek R von Schroetter C López García F. NMR solution structure of the human prion protein. Proc Natl Acad Sci USA. (2000) 97:145–50. doi: 10.1073/pnas.97.1.145, PMID: 10618385 PMC26630

[36] Oesch B Westaway D Wälchli M McKinley MP Kent SB Aebersold R. A cellular gene encodes scrapie PrP 27-30 protein. Cell. (1985) 40:735–46. doi: 10.1016/0092-8674(85)90333-2 2859120

[37] Dürig J Giese A Schulz-Schaeffer W Rosenthal C Schmücker U Bieschke J. Differential constitutive and activation-dependent expression of prion protein in human peripheral blood leucocytes. Br J Haematol. (2000) 108:488–95. doi: 10.1046/j.1365-2141.2000.01881.x, PMID: 10759704

[38] Zhang CC Steele AD Lindquist S Lodish HF. Prion protein is expressed on long-term repopulating hematopoietic stem cells and is important for their self-renewal. Proc Natl Acad Sci USA. (2006) 103:2184–9. doi: 10.1073/pnas.0510577103, PMID: 16467153 PMC1413720

[39] Isaacs JD Jackson GS Altmann DM. The role of the cellular prion protein in the immune system. Clin Exp Immunol. (2006) 146:1–8. doi: 10.1111/j.1365-2249.2006.03194.x, PMID: 16968391 PMC1809729

[40] Glier H Simak J Panigaj M Gelderman MP Vostal JG Holada K. Expression of the cellular prion protein affects posttransfusion recovery and survival of red blood cells in mice. Transfusion. (2015) 55:2590–6. doi: 10.1111/trf.13190, PMID: 26033638

[41] Risitano AM Holada K Chen G Simak J Vostal JG Young NS. CD34+ cells from paroxysmal nocturnal hemoglobinuria (PNH) patients are deficient in surface expression of cellular prion protein (PrPc). Exp Hematol. (2003) 31:65–72. doi: 10.1016/s0301-472x(02)01011-1, PMID: 12543108

[42] Zivny JH Gelderman MP Xu F Piper J Holada K Simak J. Reduced erythroid cell and erythropoietin production in response to acute anemia in prion protein-deficient (Prnp−/−) mice. Blood Cells Mol Dis. (2008) 40:302–7. doi: 10.1016/j.bcmd.2007.09.009, PMID: 17964827

[43] Paar C Wurm S Pfarr W Sonnleitner A Wechselberger C. Prion protein resides in membrane microclusters of the immunological synapse during lymphocyte activation. Eur J Cell Biol. (2007) 86:253–64. doi: 10.1016/j.ejcb.2007.03.001, PMID: 17449139

[44] Basler K Oesch B Scott M Westaway D Wälchli M Groth DF. Scrapie and cellular PrP isoforms are encoded by the same chromosomal gene. Cell. (1986) 46:417–28. doi: 10.1016/0092-8674(86)90662-8, PMID: 2873895

[45] Pan KM Baldwin M Nguyen J Gasset M Serban A Groth D. Conversion of alpha-helices into beta-sheets features in the formation of the scrapie prion proteins. Proc Natl Acad Sci USA. (1993) 90:10962–6. doi: 10.1073/pnas.90.23.10962 7902575 PMC47901

[46] Stahl N Baldwin MA Teplow DB Hood L Gibson BW Burlingame AL. Structural studies of the scrapie prion protein using mass spectrometry and amino acid sequencing. Biochemistry. (1993) 32:1991–2002. doi: 10.1021/bi00059a016 8448158

[47] Stahl N Prusiner SB. Prions and prion proteins. FASEB J. (1991) 5:2799–807. doi: 10.1096/fasebj.5.13.1916104 1916104

[48] Caughey BW Dong A Bhat KS Ernst D Hayes SF Caughey WS. Secondary structure analysis of the scrapie-associated protein PrP 27-30 in water by infrared spectroscopy. Biochemistry. (1991) 30:7672–80. doi: 10.1021/bi00245a003, PMID: 1678278

[49] Baron GS Hughson AG Raymond GJ Offerdahl DK Barton KA Raymond LD. Effect of glycans and the glycophosphatidylinositol anchor on strain dependent conformations of scrapie prion protein: improved purifications and infrared spectra. Biochemistry. (2011) 50:4479–90. doi: 10.1021/bi2003907, PMID: 21539311 PMC3101284

[50] Tanaka M Chien P Naber N Cooke R Weissman JS. Conformational variations in an infectious protein determine prion strain differences. Nature. (2004) 428:323–8. doi: 10.1038/nature02392, PMID: 15029196

[51] Requena JR Wille H. The structure of the infectious prion protein: experimental data and molecular models. Prion. (2014) 8:60–6. doi: 10.4161/pri.28368 24583975 PMC7030906

[52] Kraus A Hoyt F Schwartz CL Hansen B Artikis E Hughson AG. High-resolution structure and strain comparison of infectious mammalian prions. Mol Cell. (2021) 81:4540–4551.e6. doi: 10.1016/j.molcel.2021.08.011, PMID: 34433091

[53] Manka SW Zhang W Wenborn A Betts J Joiner S Saibil HR. 2.7 Å cryo-EM structure of ex vivo RML prion fibrils. Nat Commun. (2022) 13:4004. doi: 10.1038/s41467-022-30457-7, PMID: 35831275 PMC9279362

[54] Manka SW Wenborn A Betts J Joiner S Saibil HR Collinge J. A structural basis for prion strain diversity. Nat Chem Biol. (2023) 19:607–13. doi: 10.1038/s41589-022-01229-7, PMID: 36646960 PMC10154210

[55] Hoyt F Standke HG Artikis E Schwartz CL Hansen B Li K. Cryo-EM structure of anchorless RML prion reveals variations in shared motifs between distinct strains. Nat Commun. (2022) 13:4005. doi: 10.1038/s41467-022-30458-6, PMID: 35831291 PMC9279418

[56] Li Q Jaroniec CP Surewicz WK. Cryo-EM structure of disease-related prion fibrils provides insights into seeding barriers. Nat Struct Mol Biol. (2022) 29:962–5. doi: 10.1038/s41594-022-00833-4, PMID: 36097290 PMC9639217

[57] Kamali-Jamil R Vázquez-Fernández E Tancowny B Rathod V Amidian S Wang X. The ultrastructure of infectious L-type bovine spongiform encephalopathy prions constrains molecular models. PLoS Pathog. (2021) 17:e1009628. doi: 10.1371/journal.ppat.1009628, PMID: 34061899 PMC8195424

[58] Vázquez-Fernández E Vos MR Afanasyev P Cebey L Sevillano AM Vidal E. The structural architecture of an infectious mammalian prion using Electron Cryomicroscopy. PLoS Pathog. (2016) 12:e1005835. doi: 10.1371/journal.ppat.1005835, PMID: 27606840 PMC5015997

[59] Igel A Fornara B Rezaei H Béringue V. Prion assemblies: structural heterogeneity, mechanisms of formation, and role in species barrier. Cell Tissue Res. (2023) 392:149–66. doi: 10.1007/s00441-022-03700-2, PMID: 36399162 PMC10113350

[60] Bohl J Moudjou M Herzog L Reine F Sailer F Klute H. The smallest infectious substructure encoding the prion strain structural determinant revealed by spontaneous dissociation of misfolded prion protein assemblies. J Mol Biol. (2023) 435:168280. doi: 10.1016/j.jmb.2023.168280, PMID: 37730082

[61] Lane WJ Vege S Mah HH Lomas-Francis C Aguad M Smeland-Wagman R. Automated typing of red blood cell and platelet antigens from whole exome sequences. Transfusion. (2019) 59:3253–63. doi: 10.1111/trf.15473, PMID: 31392742

[62] Doolan KM Colby DW. Conformation-dependent epitopes recognized by prion protein antibodies probed using mutational scanning and deep sequencing. J Mol Biol. (2015) 427:328–40. doi: 10.1016/j.jmb.2014.10.024, PMID: 25451031 PMC5885637

[63] Kitamoto T Tateishi J. Human prion diseases with variant prion protein. Philos Trans R Soc Lond Ser B Biol Sci. (1994) 343:391–8. doi: 10.1098/rstb.1994.0034 7913756

[64] Beck JA Poulter M Campbell TA Adamson G Uphill JB Guerreiro R. PRNP allelic series from 19 years of prion protein gene sequencing at the MRC prion unit. Hum Mutat. (2010) 31:E1551–63. doi: 10.1002/humu.21281, PMID: 20583301

[65] Mead S Stumpf MP Whitfield J Beck JA Poulter M Campbell T. Balancing selection at the prion protein gene consistent with prehistoric kurulike epidemics. Science. (2003) 300:640–3. doi: 10.1126/science.1083320, PMID: 12690204

[66] Soldevila M Calafell F Andrés AM Yagüe J Helgason A Stefánsson K. Prion susceptibility and protective alleles exhibit marked geographic differences. Hum Mutat. (2003) 22:104–5. doi: 10.1002/humu.9157, PMID: 12815603

[67] Dimitrijević R Cadez I Keckarević-Marković M Keckarević D Kecmanović M Dobricić V. Polymorphisms of the prion protein gene (PRNP) in a Serbian population. Int J Neurosci. (2010) 120:496–501. doi: 10.3109/00207451003765907, PMID: 20583902

[68] Petraroli R Pocchiari M. Codon 219 polymorphism of PRNP in healthy Caucasians and Creutzfeldt-Jakob disease patients. Am J Hum Genet. (1996) 58:888–9. 8644754 PMC1914686

[69] Ohkubo T Sakasegawa Y Asada T Kinoshita T Goto Y Kimura H. Absence of association between codon 129/219 polymorphisms of the prion protein gene and Alzheimer's disease in Japan. Ann Neurol. (2003) 54:553–4. doi: 10.1002/ana.10748, PMID: 14520676

[70] Tsai MT Su YC Chen YH Chen CH. Lack of evidence to support the association of the human prion gene with schizophrenia. Mol Psychiatry. (2001) 6:74–8. doi: 10.1038/sj.mp.4000790, PMID: 11244488

[71] Yu SL Jin L Sy MS Mei FH Kang SL Sun GH. Polymorphisms of the PRNP gene in Chinese populations and the identification of a novel insertion mutation. Eur J Hum Genet. (2004) 12:867–70. doi: 10.1038/sj.ejhg.5201245, PMID: 15266305

[72] Jeong BH Nam JH Lee YJ Lee KH Jang MK Carp RI. Polymorphisms of the prion protein gene (PRNP) in a Korean population. J Hum Genet. (2004) 49:319–24. doi: 10.1007/s10038-004-0150-7 15148589

[73] Ahn K Kim E Kwon YA Kim DK Lee JE Jo SA. No association of prion protein gene polymorphisms with Alzheimer's disease in Korean population. Exp Mol Med. (2006) 38:727–31. doi: 10.1038/emm.2006.85, PMID: 17202849

[74] Jeong BH Lee KH Jeong YE Hwang KA Lee YJ Carp RI. Polymorphisms at codons 129 and 219 of the prion protein gene (PRNP) are not associated with sporadic Alzheimer's disease in the Korean population. Eur J Neurol. (2007) 14:621–6. doi: 10.1111/j.1468-1331.2007.01786.x 17539938

[75] Moe Lee S Ran JY Choi BY Wook Hyeon J Sun Park J Kyeong Kim C. Genotype patterns and characteristics of PRNP in the Korean population. Prion. (2012) 6:375–82. doi: 10.4161/pri.20195, PMID: 22561193 PMC3609067

[76] Prusiner SB. Prions. Proc Natl Acad Sci USA. (1998) 95:13363–83. doi: 10.1073/pnas.95.23.13363, PMID: 9811807 PMC33918

[77] Kobayashi A Teruya K Matsuura Y Shirai T Nakamura Y Yamada M. The influence of PRNP polymorphisms on human prion disease susceptibility: an update. Acta Neuropathol. (2015) 130:159–70. doi: 10.1007/s00401-015-1447-7, PMID: 26022925

[78] Kosami K Ae R Hamaguchi T Sanjo N Tsukamoto T Kitamoto T. Methionine homozygosity for PRNP polymorphism and susceptibility to human prion diseases. J Neurol Neurosurg Psychiatry. (2022) 93:779–84. doi: 10.1136/jnnp-2021-328720, PMID: 35387866

[79] Jeong BH Na HR Bae JC Lee KH Lee YJ Kim NH. Absence of association between codon 129 and 219 polymorphisms of the prion protein gene and vascular dementia. Dement Geriatr Cogn Disord. (2007) 24:86–90. doi: 10.1159/000103913, PMID: 17570906

[80] Bishop MT Pennington C Heath CA Will RG Knight RS. PRNP variation in UK sporadic and variant Creutzfeldt Jakob disease highlights genetic risk factors and a novel non-synonymous polymorphism. BMC Med Genet. (2009) 10:146. doi: 10.1186/1471-2350-10-146, PMID: 20035629 PMC2806268

[81] Bratosiewicz-Wasik J Wasik TJ Liberski PP. Codon 219 in Creutzfeldt-Jakob disease in Poland. Acta Neurobiol Exp (Wars). (2002) 62:149–51. doi: 10.55782/ane-2002-1433, PMID: 12416392

[82] Wang Y Qiao XY Zhao CB Gao X Yao ZW Qi L. Report on the first Chinese family with Gerstmann-Sträussler-Scheinker disease manifesting the codon 102 mutation in the prion protein gene. Neuropathology. (2006) 26:429–32. doi: 10.1111/j.1440-1789.2006.00704.x, PMID: 17080720

[83] Shi Q Zhou W Chen C Zhang BY Xiao K Wang Y. Rare E196A mutation in PRNP gene of 3 Chinese patients with Creutzfeldt-Jacob disease. Prion. (2016) 10:331–7. doi: 10.1080/19336896.2016.1190897, PMID: 27310471 PMC5082964

[84] Cartier RL Fernández OJ Ramírez VE. Genetic markers in four Chilean families with familial Creutzfeldt-Jakob disease. Rev Med Chile. (2006) 134:1116–22. doi: 10.4067/s0034-98872006000900005, PMID: 17171212

[85] Takayanagi M Suzuki K Nakamura T Hirata K Satoh K Kitamoto T. Genetic Creutzfeldt-Jakob disease with a glutamate-to-lysine substitution at codon 219 (E219K) in the presence of the E200K mutation presenting with rapid progressive dementia following slowly progressive clinical course. Rinsho Shinkeigaku. (2018) 58:682–7. doi: 10.5692/clinicalneurol.cn-001206, PMID: 30369528

[86] Seno H Tashiro H Ishino H Inagaki T Nagasaki M Morikawa S. New haplotype of familial Creutzfeldt-Jakob disease with a codon 200 mutation and a codon 219 polymorphism of the prion protein gene in a Japanese family. Acta Neuropathol. (2000) 99:125–30. doi: 10.1007/pl00007415, PMID: 10672318

[87] Furukawa H Kitamoto T Tanaka Y Tateishi J. New variant prion protein in a Japanese family with Gerstmann-Sträussler syndrome. Brain Res Mol Brain Res. (1995) 30:385–8. doi: 10.1016/0169-328x(95)00034-p, PMID: 7637591

[88] Tanaka Y Minematsu K Moriyasu H Yamaguchi T Yutani C Kitamoto T. A Japanese family with a variant of Gerstmann-Sträussler-Scheinker disease. J Neurol Neurosurg Psychiatry. (1997) 62:454–7. doi: 10.1136/jnnp.62.5.454, PMID: 9153600 PMC486847

[89] Wang J Xiao K Zhou W Shi Q Dong XP. Analysis of 12 Chinese patients with proline-to-leucine mutation at codon 102-associated Gerstmann-Sträussler-Scheinker disease. J Clin Neurol. (2019) 15:184–90. doi: 10.3988/jcn.2019.15.2.184, PMID: 30877692 PMC6444146

[90] Muramoto T Tanaka T Kitamoto N Sano C Hayashi Y Kutomi T. Analyses of Gerstmann-Straussler syndrome with 102Leu219Lys using monoclonal antibodies that specifically detect human prion protein with 219Glu. Neurosci Lett. (2000) 288:179–82. doi: 10.1016/s0304-3940(00)01232-5 10889337

[91] Nishida Y Sodeyama N Toru Y Toru S Kitamoto T Mizusawa H. Creutzfeldt-Jakob disease with a novel insertion and codon 219 Lys/Lys polymorphism in PRNP. Neurology. (2004) 63:1978–9. doi: 10.1212/01.wnl.0000144196.43430.e1, PMID: 15557533

[92] Barbanti P Fabbrini G Salvatore M Petraroli R Cardone F Maras B. Polymorphism at codon 129 or codon 219 of PRNP and clinical heterogeneity in a previously unreported family with Gerstmann-Sträussler-Scheinker disease (PrP-P102L mutation). Neurology. (1996) 47:734–41. doi: 10.1212/wnl.47.3.734 8797472

[93] Noguchi-Shinohara M Hamaguchi T Kitamoto T Sato T Nakamura Y Mizusawa H. Clinical features and diagnosis of dura mater graft associated Creutzfeldt Jakob disease. Neurology. (2007) 69:360–7. doi: 10.1212/01.wnl.0000266624.63387.4a, PMID: 17646628

[94] Ikawa M Yoneda M Matsunaga A Nakagawa H Kazama-Suzuki A Miyashita N. Unique clinicopathological features and PrP profiles in the first autopsied case of dura mater graft-associated Creutzfeldt-Jakob disease with codon 219 lysine allele observed in Japanese population. J Neurol Sci. (2009) 285:265–7. doi: 10.1016/j.jns.2009.07.019, PMID: 19666177

[95] Collinge J. Molecular neurology of prion disease. J Neurol Neurosurg Psychiatry. (2005) 76:906–19. doi: 10.1136/jnnp.2004.048660, PMID: 15965195 PMC1739714

[96] Lukic A Beck J Joiner S Fearnley J Sturman S Brandner S. Heterozygosity at polymorphic codon 219 in variant creutzfeldt-jakob disease. Arch Neurol. (2010) 67:1021–3. doi: 10.1001/archneurol.2010.184, PMID: 20697057

[97] Rossetti G Cong X Caliandro R Legname G Carloni P. Common structural traits across pathogenic mutants of the human prion protein and their implications for familial prion diseases. J Mol Biol. (2011) 411:700–12. doi: 10.1016/j.jmb.2011.06.008, PMID: 21689662

[98] Jahandideh S Jamalan M Faridounnia M. Molecular dynamics study of the dominant-negative E219K polymorphism in human prion protein. J Biomol Struct Dyn. (2015) 33:1315–25. doi: 10.1080/07391102.2014.945486, PMID: 25027605

[99] Jani V Sonavane U Joshi R. Detecting early stage structural changes in wild type, pathogenic and non-pathogenic prion variants using Markov state model. RSC Adv. (2019) 9:14567–79. doi: 10.1039/c9ra01507h, PMID: 35519320 PMC9064127

[100] Sigurdson CJ Nilsson KP Hornemann S Manco G Fernández-Borges N Schwarz P. A molecular switch controls interspecies prion disease transmission in mice. J Clin Invest. (2010) 120:2590–9. doi: 10.1172/jci42051, PMID: 20551516 PMC2898603

[101] Pérez DR Damberger FF Wüthrich K. Horse prion protein NMR structure and comparisons with related variants of the mouse prion protein. J Mol Biol. (2010) 400:121–8. doi: 10.1016/j.jmb.2010.04.066, PMID: 20460128

[102] Wen Y Li J Yao W Xiong M Hong J Peng Y. Unique structural characteristics of the rabbit prion protein. J Biol Chem. (2010) 285:31682–93. doi: 10.1074/jbc.M110.118844, PMID: 20639199 PMC2951240

[103] Mollica L Giachin G. Recognition mechanisms between a Nanobody and disordered epitopes of the human prion protein: an integrative molecular dynamics study. J Chem Inf Model. (2023) 63:531–45. doi: 10.1021/acs.jcim.2c01062, PMID: 36580661 PMC9875307

[104] Korth C Kaneko K Groth D Heye N Telling G Mastrianni J. Abbreviated incubation times for human prions in mice expressing a chimeric mouse-human prion protein transgene. Proc Natl Acad Sci USA. (2003) 100:4784–9. doi: 10.1073/pnas.2627989100, PMID: 12684540 PMC153633

[105] Zulianello L Kaneko K Scott M Erpel S Han D Cohen FE. Dominant-negative inhibition of prion formation diminished by deletion mutagenesis of the prion protein. J Virol. (2000) 74:4351–60. doi: 10.1128/jvi.74.9.4351-4360.2000, PMID: 10756050 PMC111952

[106] Perrier V Kaneko K Safar J Vergara J Tremblay P DeArmond SJ. Dominant-negative inhibition of prion replication in transgenic mice. Proc Natl Acad Sci USA. (2002) 99:13079–84. doi: 10.1073/pnas.182425299, PMID: 12271119 PMC130589

[107] Kaneko K Zulianello L Scott M Cooper CM Wallace AC James TL. Evidence for protein X binding to a discontinuous epitope on the cellular prion protein during scrapie prion propagation. Proc Natl Acad Sci USA. (1997) 94:10069–74. doi: 10.1073/pnas.94.19.10069, PMID: 9294164 PMC23307

[108] Telling GC Scott M Mastrianni J Gabizon R Torchia M Cohen FE. Prion propagation in mice expressing human and chimeric PrP transgenes implicates the interaction of cellular PrP with another protein. Cell. (1995) 83:79–90. doi: 10.1016/0092-8674(95)90236-8, PMID: 7553876

[109] Vogtherr M Grimme S Elshorst B Jacobs DM Fiebig K Griesinger C. Antimalarial drug quinacrine binds to C-terminal helix of cellular prion protein. J Med Chem. (2003) 46:3563–4. doi: 10.1021/jm034093h 12904059

[110] Ryou C Prusiner SB Legname G. Cooperative binding of dominant-negative prion protein to kringle domains. J Mol Biol. (2003) 329:323–33. doi: 10.1016/s0022-2836(03)00342-5, PMID: 12758079

[111] Lee CI Yang Q Perrier V Baskakov IV. The dominant-negative effect of the Q218K variant of the prion protein does not require protein X. Protein Sci. (2007) 16:2166–73. doi: 10.1110/ps.072954607, PMID: 17766375 PMC2204135

[112] Geoghegan JC Miller MB Kwak AH Harris BT Supattapone S. Trans-dominant inhibition of prion propagation in vitro is not mediated by an accessory cofactor. PLoS Pathog. (2009) 5:e1000535. doi: 10.1371/journal.ppat.1000535, PMID: 19649330 PMC2713408

[113] Perrier V Wallace AC Kaneko K Safar J Prusiner SB Cohen FE. Mimicking dominant negative inhibition of prion replication through structure-based drug design. Proc Natl Acad Sci USA. (2000) 97:6073–8. doi: 10.1073/pnas.97.11.6073, PMID: 10823951 PMC18560

[114] Crozet C Lin YL Mettling C Mourton-Gilles C Corbeau P Lehmann S. Inhibition of PrPSc formation by lentiviral gene transfer of PrP containing dominant negative mutations. J Cell Sci. (2004) 117:5591–7. doi: 10.1242/jcs.01484, PMID: 15494372 PMC2062426

[115] Shirai T Saito M Kobayashi A Asano M Hizume M Ikeda S. Evaluating prion models based on comprehensive mutation data of mouse PrP. Structure. (2014) 22:560–71. doi: 10.1016/j.str.2013.12.019, PMID: 24560805

[116] Müller-Schiffmann A Korth C. Vaccine approaches to prevent and treat prion infection: progress and challenges. BioDrugs. (2008) 22:45–52. doi: 10.2165/00063030-200822010-00005, PMID: 18215090

[117] Enari M Flechsig E Weissmann C. Scrapie prion protein accumulation by scrapie-infected neuroblastoma cells abrogated by exposure to a prion protein antibody. Proc Natl Acad Sci USA. (2001) 98:9295–9. doi: 10.1073/pnas.151242598, PMID: 11470893 PMC55414

[118] Peretz D Williamson RA Kaneko K Vergara J Leclerc E Schmitt-Ulms G. Antibodies inhibit prion propagation and clear cell cultures of prion infectivity. Nature. (2001) 412:739–43. doi: 10.1038/35089090 11507642

[119] Pankiewicz J Prelli F Sy MS Kascsak RJ Kascsak RB Spinner DS. Clearance and prevention of prion infection in cell culture by anti-PrP antibodies. Eur J Neurosci. (2006) 23:2635–47. doi: 10.1111/j.1460-9568.2006.04805.x, PMID: 16817866 PMC1779824

[120] Horiuchi M Baron GS Xiong LW Caughey B. Inhibition of interactions and interconversions of prion protein isoforms by peptide fragments from the C-terminal folded domain. J Biol Chem. (2001) 276:15489–97. doi: 10.1074/jbc.M100288200, PMID: 11279046

[121] Wang LQ Zhao K Yuan HY Li XN Dang HB Ma Y. Genetic prion disease-related mutation E196K displays a novel amyloid fibril structure revealed by cryo-EM. Sci Adv. (2021) 7:eabg9676. doi: 10.1126/sciadv.abg9676, PMID: 34516876 PMC8442898

[122] Wang LQ Zhao K Yuan HY Wang Q Guan Z Tao J. Cryo-EM structure of an amyloid fibril formed by full-length human prion protein. Nat Struct Mol Biol. (2020) 27:598–602. doi: 10.1038/s41594-020-0441-5, PMID: 32514176

[123] Heppner FL Musahl C Arrighi I Klein MA Rülicke T Oesch B. Prevention of scrapie pathogenesis by transgenic expression of anti-prion protein antibodies. Science. (2001) 294:178–82. doi: 10.1126/science.1063093, PMID: 11546838

[124] Sigurdsson EM Brown DR Daniels M Kascsak RJ Kascsak R Carp R. Immunization delays the onset of prion disease in mice. Am J Pathol. (2002) 161:13–7. doi: 10.1016/s0002-9440(10)64151-x, PMID: 12107084 PMC1850699

[125] White AR Enever P Tayebi M Mushens R Linehan J Brandner S. Monoclonal antibodies inhibit prion replication and delay the development of prion disease. Nature. (2003) 422:80–3. doi: 10.1038/nature01457, PMID: 12621436

[126] Buchholz CJ Bach P Nikles D Kalinke U. Prion protein-specific antibodies for therapeutic intervention of transmissible spongiform encephalopathies. Expert Opin Biol Ther. (2006) 6:293–300. doi: 10.1517/14712598.6.3.293, PMID: 16503737

[127] Roettger Y Du Y Bacher M Zerr I Dodel R Bach JP. Immunotherapy in prion disease. Nat Rev Neurol. (2013) 9:98–105. doi: 10.1038/nrneurol.2012.258 23247613

[128] Bard F Cannon C Barbour R Burke RL Games D Grajeda H. Peripherally administered antibodies against amyloid beta-peptide enter the central nervous system and reduce pathology in a mouse model of Alzheimer disease. Nat Med. (2000) 6:916–9. doi: 10.1038/78682 10932230

[129] Donofrio G Heppner FL Polymenidou M Musahl C Aguzzi A. Paracrine inhibition of prion propagation by anti-PrP single-chain Fv miniantibodies. J Virol. (2005) 79:8330–8. doi: 10.1128/jvi.79.13.8330-8338.2005, PMID: 15956578 PMC1143714

[130] Yu YJ Zhang Y Kenrick M Hoyte K Luk W Lu Y. Boosting brain uptake of a therapeutic antibody by reducing its affinity for a transcytosis target. Sci Transl Med. (2011) 3:84ra44. doi: 10.1126/scitranslmed.3002230, PMID: 21613623

[131] Pardridge WM Boado RJ. Reengineering biopharmaceuticals for targeted delivery across the blood-brain barrier. Methods Enzymol. (2012) 503:269–92. doi: 10.1016/b978-0-12-396962-0.00011-2, PMID: 22230573

[132] Pardridge WM. Blood-brain barrier delivery for lysosomal storage disorders with IgG-lysosomal enzyme fusion proteins. Adv Drug Deliv Rev. (2022) 184:114234. doi: 10.1016/j.addr.2022.114234, PMID: 35307484

[133] Antonyuk SV Trevitt CR Strange RW Jackson GS Sangar D Batchelor M. Crystal structure of human prion protein bound to a therapeutic antibody. Proc Natl Acad Sci USA. (2009) 106:2554–8. doi: 10.1073/pnas.0809170106, PMID: 19204296 PMC2637903

[134] Mead S Khalili-Shirazi A Potter C Mok T Nihat A Hyare H. Prion protein monoclonal antibody (PRN100) therapy for Creutzfeldt-Jakob disease: evaluation of a first-in-human treatment programme. Lancet Neurol. (2022) 21:342–54. doi: 10.1016/s1474-4422(22)00082-5, PMID: 35305340

[135] Houston F Foster JD Chong A Hunter N Bostock CJ. Transmission of BSE by blood transfusion in sheep. Lancet. (2000) 356:999–1000. doi: 10.1016/s0140-6736(00)02719-7 11041403

[136] Hunter N Foster J Chong A McCutcheon S Parnham D Eaton S. Transmission of prion diseases by blood transfusion. J Gen Virol. (2002) 83:2897–905. doi: 10.1099/0022-1317-83-11-2897 12388826

[137] Cervenakova L Yakovleva O McKenzie C Kolchinsky S McShane L Drohan WN. Similar levels of infectivity in the blood of mice infected with human-derived vCJD and GSS strains of transmissible spongiform encephalopathy. Transfusion. (2003) 43:1687–94. doi: 10.1046/j.0041-1132.2003.00586.x, PMID: 14641865

[138] Llewelyn CA Hewitt PE Knight RS Amar K Cousens S Mackenzie J. Possible transmission of variant Creutzfeldt-Jakob disease by blood transfusion. Lancet. (2004) 363:417–21. doi: 10.1016/s0140-6736(04)15486-x 14962520

[139] Tattum MH Jones S Pal S Khalili-Shirazi A Collinge J Jackson GS. A highly sensitive immunoassay for the detection of prion-infected material in whole human blood without the use of proteinase K. Transfusion. (2010) 50:2619–27. doi: 10.1111/j.1537-2995.2010.02731.x 20561299

[140] Soutyrine A Huang H Andrievskaia O Walther I Mitchell G. A novel approach for scrapie-associated prion (PrP(Sc)) detection in blood using the competitive affinity of an aggregate-specific antibody and streptavidin to PrP(Sc). Res Vet Sci. (2017) 113:115–21. doi: 10.1016/j.rvsc.2017.09.007, PMID: 28942337

[141] Carmona P Monleón E Monzón M Badiola JJ Monreal J. Raman analysis of prion protein in blood cell membranes from naturally affected scrapie sheep. Chem Biol. (2004) 11:759–64. doi: 10.1016/j.chembiol.2004.04.005, PMID: 15217609

[142] Thackray AM Ryder SJ Bujdoso R. Modification of blood cell PrP epitope exposure during prion disease. Biochem J. (2005) 390:563–71. doi: 10.1042/bj20050571, PMID: 15885031 PMC1198936

[143] Hill AF Zeidler M Ironside J Collinge J. Diagnosis of new variant Creutzfeldt-Jakob disease by tonsil biopsy. Lancet. (1997) 349:99–100. doi: 10.1016/s0140-6736(97)24002-x 8996424

[144] Hill AF Butterworth RJ Joiner S Jackson G Rossor MN Thomas DJ. Investigation of variant Creutzfeldt-Jakob disease and other human prion diseases with tonsil biopsy samples. Lancet. (1999) 353:183–9. doi: 10.1016/s0140-6736(98)12075-5, PMID: 9923873

[145] Collinge J Whitfield J McKintosh E Beck J Mead S Thomas DJ. Kuru in the 21st century--an acquired human prion disease with very long incubation periods. Lancet. (2006) 367:2068–74. doi: 10.1016/s0140-6736(06)68930-7 16798390

[146] Morales R Buytaert-Hoefen KA Gonzalez-Romero D Castilla J Hansen ET Hlavinka D. Reduction of prion infectivity in packed red blood cells. Biochem Biophys Res Commun. (2008) 377:373–8. doi: 10.1016/j.bbrc.2008.09.141, PMID: 18851948 PMC2606671

[147] Sowemimo-Coker S Kascsak R Kim A Andrade F Pesci S Kascsak R. Removal of exogenous (spiked) and endogenous prion infectivity from red cells with a new prototype of leukoreduction filter. Transfusion. (2005) 45:1839–44. doi: 10.1111/j.1537-2995.2005.00640.x, PMID: 16371036

[148] Anstee DJ. Prion protein and the red cell. Curr Opin Hematol. (2007) 14:210–4. doi: 10.1097/MOH.0b013e3280d2b757 17414209

[149] Cahill MR Murphy T Khan M Fagan J Murphy WG. Phase I/II safety study of transfusion of prion-filtered red cell concentrates in transfusion-dependent patients. Vox Sang. (2010) 99:174–6. doi: 10.1111/j.1423-0410.2010.01330.x, PMID: 20345513

[150] Sivakumaran M. Transmission of BSE by blood transfusion. Lancet. (2000) 356:1771–2. doi: 10.1016/s0140-6736(05)71967-x 11095288

[151] Harris DA. Cellular biology of prion diseases. Clin Microbiol Rev. (1999) 12:429–44. doi: 10.1128/cmr.12.3.429, PMID: 10398674 PMC100247

[152] Brown P. BSE and transmission through blood. Lancet. (2000) 356:955–6. doi: 10.1016/s0140-6736(00)02706-9 11041390

